# Relationship between sleep quality and dizziness

**DOI:** 10.1371/journal.pone.0192705

**Published:** 2018-03-07

**Authors:** Sung Kyun Kim, Ji Hoon Kim, Seung Sik Jeon, Seok Min Hong

**Affiliations:** Department of Otorhinolaryngology-Head and Neck Surgery, Dongtan Sacred Heart Hospital, Hallym University College of Medicine, Hwaseong, Korea; Charite Medical University Berlin, GERMANY

## Abstract

**Objective:**

Poor sleep quality has a number of significant negative effects on daytime function. However, few studies have examined sleep quality in patients with dizziness. Here, we investigated the potential association between sleep quality and various types of dizziness.

**Subjects and methods:**

We examined dizziness and sleep disturbance in 237 patients experiencing dizziness using Korean versions of the Pittsburgh Sleep Quality Index (PSQI), the Insomnia Severity Index (ISI), and Dizziness Handicap Inventory (DHI). All participants were classified as having benign paroxysmal positional vertigo (BPPV), Ménière’s disease (MD), vestibular neuritis (VN), vestibular migraine (VM), psychogenic dizziness (PD), or Other.

**Results:**

The mean PSQI and ISI scores were highest in the PD group. The rate of sleep disturbance was highest in the Other group when the cut-off score for each questionnaire was set differently, except ISI ≥ 15. The correlation between DHI and sleep disturbance indices was highest in the VM group. Multivariate regression showed that PSQI score and DHI-E score were significantly related to the PD and Other groups, while the Other group was significantly related to the ISI score.

**Conclusion:**

The findings of this study strongly suggest that there are associations between sleep quality and some disease subtypes associated with dizziness. Therefore, it is important to consider sleep disturbance in patients with psychogenic dizziness, such as phobic postural vertigo and chronic subjective dizziness, or nonspecific dizziness.

## Introduction

Dizziness is a nonspecific sensation of disorientation or impairment in spatial perception and stability. It is a common complaint in the primary care setting, the emergency department, and clinics specializing in dizziness requiring medical attention [1]. Many clinical conditions are associated with dizziness.

Among the factors that affect the quality of life in patients with dizziness, anxiety and depressive mood sleep disturbance can affect clinical presentations and therapeutic outcomes [2]. Sleep disturbance, particularly insomnia, can cause various psychiatric and physical health problems. Poor sleep quality has also been considered a strong risk factor of psychiatric illness, such as anxiety disorder, major depressive disorder [3], and associated with many types of metabolic disease [4], obstructive airway disease, and cancer [5]. Despite the many medical issues arising from sleep disturbance, there have been few studies of this issue in cases of vestibular disorder. Recent studies have investigated the relationship between sleep disturbance and dizziness. The severities of dizziness and sleep-related problems are closely related to each other, as determined through validated questionnaires, such as the Pittsburgh Sleep Quality Index (PSQI) [6], and these two issues are also correlated with poor quality of life and emotional stress [7].

Gomez *et al*. [8] reported that sleep deprivation for 36 h affected postural ability and attention-demanding tasks as detained by posturography and calf vibration in healthy controls. In a study of vestibular function in patients with obstructive sleep apnea syndrome (OSAS), the OSAS group had a higher rate of abnormalities on caloric test, increased saccadic eye movement latency, and changes in smooth-pursuit movements [9]. Sugaya *et al*. [10] and Nakayama *et al*. [11] also investigated the relationship between dizziness and sleep disturbance, but these studies focused only on the comorbidity between sleep disturbance and chronic dizziness or one type of disease.

In the present study, we investigated the incidences of sleep disturbance indices in a relatively large sample of patients complaining of dizziness, to determine whether there are differences in the occurrence of sleep disturbance among types of vestibular disease using comprehensive questionnaires. This may help us to understand more fully the connection between sleep quality and vestibular dysfunction, and gain insight into the potential pathogenesis of vestibular diseases.

## Materials and methods

This cross-sectional study was conducted in patients experiencing dizziness. Ethical approval was obtained from the Hallym University Institutional Review Board. All patients provided informed consent to participation in the study. All steps of the study were conducted in accordance with the Declaration of Helsinki on biomedical studies involving human subjects.

### Subjects and procedures

The study was carried out as a cross-sectional survey between January 2013 and November 2015. All patients presenting to the Otolaryngology outpatient department with dizziness as their primary complaint were asked to participate in this study. Patients with central vertigo, a history of previous sleep-related disorder, or confirmed psychological disorder were excluded.

All participants underwent a detailed head and neck neurotological examination. Vestibular function tests included positional, positioning maneuvers, caloric test, and pursuit, saccadic, and optokinetic tests using a video-based system. In addition, audiological tests (pure tone audiometry and speech audiometry), a complete blood count, and blood chemistry tests (fasting blood sugar, urea nitrogen, creatinine, electrolytes, and liver function tests) were performed. If further evaluations were required, we performed vestibular evoked myogenic potential, electrocochleography, computed tomography, or magnetic resonance imaging to reach the final diagnosis.

Patients were divided into the benign paroxysmal positional vertigo (BPPV), vestibular neuritis (VN), Ménière’s disease (MD), vestibular migraine (VM), psychogenic dizziness (PD), and Other groups. The psychogenic dizziness group included patients with chronic subjective dizziness and phobic positional vertigo. Dizziness after traumatic brain injury (TBI), sudden sensorineural hearing loss with vertigo, nonspecific dizziness, and bilateral vestibulopathy comprised the Other group.

### Assessment of vestibular symptoms and sleep disturbance

Evaluation of sleep disturbance was conducted using the Korean version of the PSQI and Insomnia Severity Index (ISI). These scales were completed at the first visit. The Korean versions of these scales were translations of the original English text into Korean. The degrees of severity of dizziness were evaluated using the Korean version of the Dizziness Handicap Inventory (DHI).

PSQI is a self-rated questionnaire about sleep quality during the last 1 month that consists of 18 items with a 4-point Likert-type response format [12]. PSQI contains a 0–3 interval scale with seven components (subjective sleep quality, sleep latency, sleep duration, habitual sleep efficiency, sleep difficulty, use of sleeping medication, and difficulty of diurnal awakening). Buysse *et al*. [13] reported that the English version of the PSQI is highly reliable (Cronbach’s α coefficient = .83). The sensitivity and specificity were 89.6%, 86.5% for patients with sleep disorder, using a cut-off score of 5. The Korean version of PSQI also showed high reliability (Cronbach’s α coefficient = .84), but the best cut-off point was 8.5 [14].

ISI measures sleep maintenance difficulties, satisfaction with current sleep patterns, interference with daily functioning, noticeable impairment attributed to the sleep problem, and the degree of concern caused by the sleep problem with seven questions [15]. The total score ranges from 0 (no disability) to 28 (severe disability) and is rated from 0 (not at all satisfied) to 4 (very much satisfied) for each item. A cutoff score of 15 has been used as the threshold for clinically significant insomnia and a score between 8 and 14 has been considered sub-threshold insomnia [15].

The DHI is a 25-item questionnaire addressing the self-perceived handicapping effects of vestibular disease in terms of the patient’s quality of life emotionally, physically, and functionally [16]. The total score ranges from 0 (no handicap) to 100 (severe handicap), and each score is also evaluated separately.

### Statistical analysis

Collected data were analyzed using SPSS version 22.0 (SPSS Inc., Chicago, IL). The data are presented as the means ± standard deviation, and *p* < 0.05 was set as the threshold for statistical significance. For statistical comparisons, all six groups (BPPV, VN, MD, VM, PD, and Other groups) were compared against each other using two-way ANOVA and Bonferroni’s *post hoc* test. The cut-off values of PSQI and ISI were set by referring to the previous study [13–15]. The chi-square test was used to test the difference in sleep disturbance between the groups. Pearson’s correlation analyses were used to examine the relationship between DHI and sleep disturbance. Multiple regression analyses were utilized to examine whether each factor, including types of disease, were related to PSQI and ISI.

## Results

This study was performed in a population of 237 patients with dizziness. The mean age of the patients was 41.73 ± 12.77 years (range 11–78 years) and the male/female ratio was 78:169. Seventy-four patients (26 males and 48 females) had BPPV, 42 (13 males and 29 females) had MD, 36 (12 males and 24 females) had VN, 54 (9 males and 45 females) had VM, 20 (8 males and 12 females) were classified into the PD group, and the remaining 21 patients (10 males and 11 females) were classified into the Other group (Table 1).

**Table 1:** Demographic characteristics of the patients.

Parameters	*n* (%)*
Age (years)	41.73 ± 12.77 (11–78)
Male:Female	78:169
Cause of dizziness	
BPPV	74 (31.2)
Ménière’s disease	32 (13.5)
Vestibular neuritis	36 (15.2)
Vestibular migraine	54 (22.8)
Psychogenic dizziness	20 (8.4)
Other	21 (8.9)
Total	237 (100)

*Unless otherwise indicated.

BPPV, benign paroxysmal positional vertigo.

**Fig 1:**
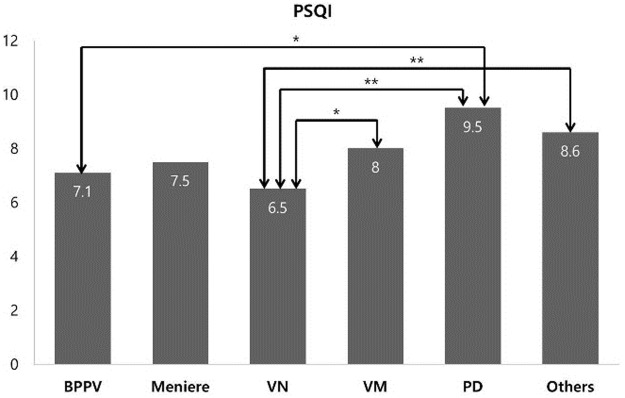
The mean PSQI scores for each group. The mean PSQI score of all subjects was 7.9 and was highest in the PD group (*p < 0.05, **p < 0.01).

**Fig 2:**
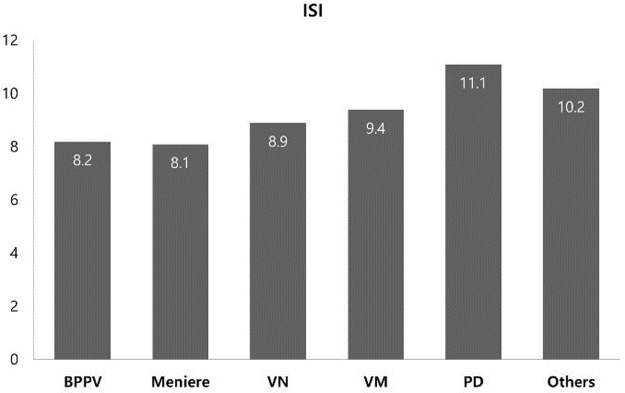
The mean ISI scores for each group. The mean ISI score of all subjects was 9.3 and was highest in the PD group, similar to PSQI. However, there were no significant differences between the groups.

Table 2 shows the mean PSQI and ISI scores and ratio of sleep disturbance compared by setting various cut-off values in each group. PSQI and ISI scores were highest in the PD group. The PSQI score of the PD group was significantly different to those of the BPPV (*p* < 0.05) and VN (*p* < 0.01) groups. There was also a associated with PSQI score between the Other group and VN group (*p* < 0.01), and between the VN and VM groups (*p* < 0.05) (Fig 1). However, there were no significant differences in ISI score among the groups (Fig 2). The sleep disturbance ratio of each group varied with changes in the cut-off value of the scores. In the other three cut-off value categories, except for clinical insomnia (15 ≥ ISI), the sleep disturbance ratio of the Other group was the highest. PSQI > 5 (*p* = 0.006) and ISI ≥ 8 (*p* = 0.027) were associated with sleep disturbance in each disease, as determined by chi-square analysis based on sleep disturbance according to various cut-off values.

**Table 2:** Distribution of patients with high PSQI and ISI scores and changes in the sleep disturbance ratio after applying different cut-off values.

	Mean PSQI/ISI	PSQI		ISI	
		PSQI > 5 *n*/total (%)	PSQI > 8.5 *n*/total (%)	ISI ≥ 8 *n*/total (%)	ISI ≥ 15 *n*/total (%)
BPPV	7.1/8.2	45/74 (60.8)	26/74 (35.1)	36/74 (48.6)	12/74 (16.2)
MD	7.5/8.1	20/32 (62.5)	12/32 (37.5)	12/32 (37.5)	6/32 (18.8)
VN	6.5/8.9	19/36 (52.8)	9/36 (25.0)	19/36 (52.8)	5/36 (13.9)
VM	8.0/9.4	40/54 (74.1)	26/54 (48.1)	33/54 (61.1)	14/54 (25.9)
PD	9.5/11.1	18/20 (90.0)	10/20 (50.0)	13/20 (65.0)	7/20 (35.0)
Other	8.6/10.2	19/21 (90.5)	11/21 (52.4)	17/21 (81.0)	3/21 (14.3)
Total	7.9/9.3	161/237 (67.9)	93/237 (39.2)	126/237 (53.2)	47/237 (19.8)
*p*		0.006^†^	0.206	0.027*	0.310

The relationships between DHI, PSQI, and ISI scores used in this study were examined in each group (Table 3). The correlations between the DHI score and the PSQI and ISI scores were highest in the VM group. In the BPPV and VM groups, PSQI and ISI scores showed statistically significant correlations with DHI. In the VN group, only PSQI showed a significant correlation with DHI.

**Table 3:** Correlation coefficient between DHI and the tools used for measuring sleep disturbance in each group.

		BPPV		MD		VN		VM		PD		Other	
		PSQI	ISI	PSQI	ISI	PSQI	ISI	PSQI	ISI	PSQI	ISI	PSQI	ISI
DHI	PCC	0.269	0.306	0.293	0.143	0.330	0.235	0.491	0.415	0.176	0.113	0.142	0.165
	*p*	0.021*	0.008^†^	0.104	0.180	0.049*	0.168	0.000^†^	0.002^†^	0.458	0.636	0.409	0.336

*p < 0.05.

^†^*p* < 0.01.

Logistic regression analyses were conducted with age, sex, DHI subscales, and the types of diseases as independent variables (Tables 4 and 5). Multivariate regression analyses showed that PD (*p* < 0.05, β = 1.820) and Other (*p* < 0.01, β = 2.262) groups were significantly related to PSQI score and DHI-E score (*p* < 0.05, β = 0.195), and the Other (*p* < 0.05, β = 3.237) group showed a significant relation to the ISI score.

**Table 4:** Multiple regression analyses of PSQI (a) in relation to subscales of DHI, age, sex, and all groups.

Predictive Variables	Regression Coefficient	Standard error	Standardized Regression Coefficient	*p*-value	VIF
Age	0.019	0.018	0.070	0.287	1.155
Sex	0.257	0.472	0.034	0.587	1.077
DHI-P	–0.018	0.057	–0.027	0.756	2.081
DHI-E	0.096	0.049	0.199	0.050	2.768
DHI-F	0.069	0.048	0.163	0.158	3.593
BPPV					
MD	0.559	0.713	0.054	0.434	1.288
VN	–0.612	9.677	–0.062	0.367	1.283
VM	1.037	0.624	0.123	0.098	1.487
PD	1.820	0.842	0.143	0.032*	1.191
Other	2.262	0.830	0.182	0.007†	1.208

*p < 0.05.

^†^*p* < 0.01.

R = 0.405, R^2^ = 0.164, adjusted R^2^ = 0.127. VIF, variance inflation factor.

**Table 5:** Multiple regression analyses of ISI in relation to subscales of DHI, age, sex, and all groups.

Predictive Variables	Regression Coefficient	Standard error	Standardized Regression Coefficient	*p*-value	VIF
Age	0.030	0.033	0.061	0.363	1.155
Sex	1.093	0.854	0.083	0.202	1.077
DHI-P	–0.005	0.103	–0.004	0.965	2.081
DHI-E	0.195	0.088	0.229	0.029*	2.768
DHI-F	0.064	0.087	0.087	0.463	3.593
BPPV					
MD	0.204	1.288	0.011	0.875	1.288
VN	0.561	1.224	0.032	0.647	1.283
VM	1.225	1.128	0.083	0.279	1.487
PD	1.993	1.523	0.089	0.192	1.191
Other	3.237	1.500	0.148	0.032*	1.208

*p < 0.05.

R = 0.347, R^2^ = 0.120, adjusted R^2^ = 0.081.

## Discussion

The relationship between sleep disturbance and dizziness has recently begun to be reported. Quality of sleep has become important [17], and attempts have been made to determine its relationships with various disease entities [10,18]. Konomi reported that the PSQI score was higher in psychogenic dizziness and autonomic imbalance groups in a study performed in a cohort of 52 patients with various vestibular diseases [6]. In addition, the rate of sleep disturbance in dizzy patients is higher in women, and it is also associated with emotional stress and anxiety [10]. However, these studies discussed relationships between sleep disturbance and dizziness symptoms, or only one disease entity, rather than various disease categories [11]. A study of the relationship between sleep quality and vestibular function in patients with OSAS indicated that vestibular function was decreased in those with moderate to severe OSAS [19].

Sleep disturbance is a term for subjective symptoms that may have a number of physical and psychological causative factors. Sleep disturbance has been used as a concept related to insomnia, and only studies using self-reported instruments have been reported. Recently, however, polysomnography has been used to investigate the relationships between sleep apnea and various diseases. This study focused on insomnia rather than sleep apnea or daytime somnolence and, unlike previous studies, we examined the relations of sleep quality with various vestibular diseases.

The cut-off points of PSQI and ISI in this study were set after reviewing several references. Sohn *et al*. concluded that the cut-off point was 8.5 (sensitivity 0.943, specificity 0.844) in the validation test for the Korean version of the PSQI [14]. However, their study was the only validation test of the Korean version of the PSQI. The cut-off point of the PSQI translated into various languages was 5 [20–22] and the study using the Korean version of the PSQI still sets the standard for poor sleep quality to 5. Therefore, we applied two cut-off scores in this study [23,24].

Our results indicated the relation of various vestibular diseases with sleep disturbance. In multiple regression analyses, the PD and Other groups had higher regression coefficients among factors affecting PSQI score, and the relations were statistically significant. In addition, DHI-E and the Other group were significantly associated with ISI.

PSQI and ISI scores were higher in the PD group than in the other groups in the present study. The sleep disturbance ratio of the PD group and the Other group were higher than those in the other four groups, with the exception of ISI > 15 (clinical insomnia), with various cut-off values for each measurement tool.

The PD group included patients with chronic subjective dizziness and phobic postural vertigo, which may be associated with psychological distress, including anxiety. Psychological distress and daily life stress are causally related to sleep-related experience and also show relationships with anxiety and depressive mood [25]. Therefore, this group should have high PSQI and ISI scores.

Diseases belonging to the PD group are now referred to as functional dizziness or persistent postural-perceptual dizziness (PPPD). However, research regarding these diseases is still in the early stages, and the physiological processes of diseases must be examined through postural control, visual perception, and central vestibular pathways. Thus, determination of the relationships with sleep disturbance will provide a basis for further studies.

The Other group had the highest rate of patients with dizziness associated with TBI (*n* = 8, 38.1%), followed by bilateral vestibulopathy (*n* = 5, 23.8%), and nonspecific dizziness (*n* = 5, 23.8%). Sleep disturbance is a common complication following TBI [26]. PSQI and ISI scores were higher in the Other group in this study because of the large number of patients with dizziness associated with TBI. Although injury to sleep-regulating centers in brain may be the primary factor, the connections with specific brain structures have not been established. The marked circadian rhythmicity of temperature in patients with bilateral vestibulopathy is supported by the hypothesis that vestibular input may play a role in the circadian clock [27].

However, the Other group includes several conditions that cause dizziness, and the proportion of each disease is small compared to all cases. Therefore, although the PSQI and ISI scores of the Other group are high, not all diseases belonging to the Other group are associated with sleep disturbance.

The correlation between degree of sleep disturbance and DHI was highest in the VM group. There have been few studies regarding the direct association between vestibular migraine and sleep. However, previous studies indicating the tight anatomical and functional connections as well as interactions between the vestibular system and the brain provide insight into the relationship between the VM and sleep disturbance [28,29]. VM patients showed increased signaling of the vestibular nuclei and nociceptive system compared to the control group, and reports of the trigeminovascular reflex activation in the inner ear suggest that stressful situations, such as sleep disturbance, may be precipitating factors of VM [30]. As the VM shares a significant portion of the pathophysiology of migraine, it may also be related to the correlation between migraine and sleep. Analysis of sleep microarchitecture by polysomnography indicated decreased cortical arousal activity of the rapid eye movement (REM) sleep period and reduced cyclic alternating pattern in the non-REM sleep period in patients with chronic migraine. These observations may support the pathophysiological hypothesis that sleep quality in migraine is affected by changes in the serotonergic pathway connected to the thalamocortical circuit [31,32].

In our study, some vestibular diseases were related to low sleep quality in some patients. It is important to investigate the effects of vestibular disease on sleep disturbance, which should be studied with long-term follow-up of patients.

Based on previous studies, it seems to be more helpful to understand the relationship between sleep disturbance and vestibular disease in terms of a circulatory loop than a unidirectional effect.

Our results are more meaningful than those of previous studies because we assessed the quality of sleep in patients with various vestibular diseases. However, there are analytical limitations because detailed factors were not unified due to the cross-sectional nature of the study. We did not control for the duration of illness or sex of the patients participating in the study. The average duration of BPPV was 2.2 days and the VN was 2.0 days. However, the symptom durations of the MD, VM, and Other groups varied widely among patients.

Further research is needed involving vestibular neurophysiological studies of diseases that have recently begun to attract attention, such as VM and PPPD.

## Conclusion

We found that sleep disturbance is related to some vestibular diseases. The scores of indices of sleep disturbance were higher in PD, Other, and VM groups, and they were most closely related to indices of dizziness. These observations suggested that evaluation of sleep quality is essential for assessing patients with these diseases.

## Supporting information

S1 File Data for patients. (XLS)
